# Supplementary material for: Using a health equity lens to measure patient experiences of care in diverse health care settings
Source: PLoS One. 2024 Jun 6;19(6):e0297721. doi: 10.1371/journal.pone.0297721 (PMC11156339; doi:10.1371/journal.pone.0297721)
Supplement: S1 Table — A final formatted version of the EOHCS–Ongoing, including scoring information. (DOCX) [file pone.0297721.s001.docx]

***Equity-Oriented Health Care Scale – Ongoing (EOHCS – Ongoing)^[[1]](#footnote-1)^***

| These questions ask about your experiences with staff at this service site^[[2]](#footnote-2)^ in the past 12 months^[[3]](#footnote-3)^. By staff, we mean anyone who works here including health care providers, reception staff, and others. | | | | | |
| --- | --- | --- | --- | --- | --- |
| In the past 12 months, how often did the health care providers here: | **Never** (0) | **Rarely** (1) | **Sometimes** (2) | **Usually** (3) | **Always** (4) |
| 1. Encourage you to come and see them or call when you need to? | ⃝ | ⃝ | ⃝ | ⃝ | ⃝ |
| 1. Try to make you feel as comfortable as possible? | ⃝ | ⃝ | ⃝ | ⃝ | ⃝ |
| 1. Seem open to talking about sensitive issues, for example, grief, mental health problems, substance use, or abuse experiences? | ⃝ | ⃝ | ⃝ | ⃝ | ⃝ |
| 1. Ask you about who is important in your life? | ⃝ | ⃝ | ⃝ | ⃝ | ⃝ |
| 1. Ask about basic resources that affect your health, such as food, clothing, or shelter? | ⃝ | ⃝ | ⃝ | ⃝ | ⃝ |
| 1. Give you health advice that is suitable for your everyday life? | ⃝ | ⃝ | ⃝ | ⃝ | ⃝ |
| 1. Have a negative attitude toward people using services because of mental health concerns?^[[4]](#footnote-4)^ | ⃝ | ⃝ | ⃝ | ⃝ | ⃝ |

| In the past 12 months, how often did your health care providers here: | N/A: did not have any barriers | Never (0) | Rarely (1) | Sometimes (2) | Usually (3) | Always (4) |
| --- | --- | --- | --- | --- | --- | --- |
| 1. Help you to work on any barriers you have accessing health care (e.g., costs of medication or services, problems with transportation or childcare, problems getting a referral, etc.)? | ⃝ | ⃝ | ⃝ | ⃝ | ⃝ | ⃝ |

| In the past 12 months, how often did your health care providers here: | N/A: did not need other services | Never (0) | Rarely (1) | Sometimes (2) | Usually (3) | Always (4) |
| --- | --- | --- | --- | --- | --- | --- |
| 1. Try to help you to get services that are not offered here (such as social assistance, disability benefits, housing, or parenting support)? | ⃝ | ⃝ | ⃝ | ⃝ | ⃝ | ⃝ |

| In the past 12 months: | Never (0) | Rarely (1) | Sometimes (2) | Usually (3) | Always (4) |
| --- | --- | --- | --- | --- | --- |
| 1. How often have you felt discriminated against by staff here, including health care providers, receptionists and others?^3^ | ⃝ | ⃝ | ⃝ | ⃝ | ⃝ |
| 1. How often did the staff here welcome you when you came for care? | ⃝ | ⃝ | ⃝ | ⃝ | ⃝ |
| 1. How often did staff here treat you with courtesy and respect? | ⃝ | ⃝ | ⃝ | ⃝ | ⃝ |

1. The *EOHCS – Ongoing* total score is a count of the number of items rated by patients as “always” occurring (for 10 positively worded items) and “never” occurring (for two negatively worded items), with a range of 0 to 12. Scores on the *EOHCS – Ongoing* provide an index of the degree or level of equity-oriented health care (EOHC), from lower to higher. [↑](#footnote-ref-1)
2. The *EOHCS – Ongoing* was developed in and for primary health care settings but may be appropriate for a variety of settings and contexts. We invite patients to comment on their overall experiences of care involving all staff, vs. their impressions of any one particular staff member, realizing that primary care settings are oriented to providing team-based care. [↑](#footnote-ref-2)
3. The time frame may need to be adjusted to fit the intended use. [↑](#footnote-ref-3)
4. This item is reverse scored, where never (4), rarely (3), sometimes (2), usually (1), always (0). [↑](#footnote-ref-4)
