# Supplementary material for: Using a health equity lens to measure patient experiences of care in diverse health care settings
Source: PLoS One. 2024 Jun 6;19(6):e0297721. doi: 10.1371/journal.pone.0297721 (PMC11156339; doi:10.1371/journal.pone.0297721)
Supplement: S2 Table — A final formatted version of the EOHCS–Episodic, including scoring information. (DOCX) [file pone.0297721.s002.docx]

***Equity-Oriented Health Care Scale – Episodic (EOHCS – Episodic)^[[1]](#footnote-1)^***

| **During this visit, did staff:** | **Yes** (1) | **No** (0) |
| --- | --- | --- |
| 1. **make you feel welcome?** |  |  |
| 1. **try to make you as comfortable as possible?** |  |  |
| 1. **treat you with courtesy and respect?** |  |  |
| 1. **discriminate against you?^[[2]](#footnote-2)^** |  |  |
| 1. **seem open to talking about what is important to you?** |  |  |
| 1. **learn enough about you to give useful advice?** |  |  |
| 1. **give you advice that is suitable for you?** |  |  |
| 1. **learn about problems you might have getting services (e.g., costs, transportation, getting a referral, etc.)?** |  |  |
| 1. **try to help you get services you need?** |  |  |

1. The *EOHCS – Episodic* total score is a count of the number of items rated by patients as “yes” (1) for all items except “discriminated against you” which received 1 point in the count for “no” responses, with a range of 0 to 9. Scores on the *EOHCS – Episodic* provide an index of the degree or level of equity-oriented health care (EOHC), from lower to higher. [↑](#footnote-ref-1)
2. This item is reverse scored, where Yes (0) and No (1). [↑](#footnote-ref-2)
